# Comparative Assessment of the Pharmacovigilance Systems within the Neglected Tropical Diseases Programs in East Africa—Ethiopia, Kenya, Rwanda, and Tanzania

**DOI:** 10.3390/ijerph18041941

**Published:** 2021-02-17

**Authors:** Abbie Barry, Sten Olsson, Christabel Khaemba, Joseph Kabatende, Tigist Dires, Adam Fimbo, Omary Minzi, Emile Bienvenu, Eyasu Makonnen, Appolinary Kamuhabwa, Margaret Oluka, Anastasia Guantai, Eugène van Puijenbroek, Ulf Bergman, Alex Nkayamba, Michael Mugisha, Parthasarathi Gurumurthy, Eleni Aklillu

**Affiliations:** 1 Division of Clinical Pharmacology, Department of Laboratory Medicine, Karolinska Institute, Karolinska University Hospital Huddinge, 141 86 Stockholm, Sweden; abbie.barry@ki.se (A.B.); stenolssonpv@gmail.com (S.O.); ulfkabergman@gmail.com (U.B.); 2 Pharmacy and Poisons Board, Kenya Lenana Road, P.O. Box 27663-00506 Nairobi, Kenya; cnkhaemba@gmail.com; 3 Rwanda Food and Drugs Authority, Nyarutarama Plaza, KG 9 Avenue Kigali, Rwanda; josephkabatende@gmail.com; 4 Ethiopian Food and Drug Authority, Africa Avenue, Kirkos Sub City, P.O. Box 5681 Addis Ababa, Ethiopia; tigistdires@gmail.com; 5 Tanzania Medicines and Medical Devices Authority, Off Mandela Road, Mabibo, P.O. Box 77150 Dar Es Salaam, Tanzania; adamfimbo@gmail.com (A.F.); alexnkayamba@yahoo.com (A.N.); 6 Department of Clinical Pharmacy and Pharmacology, School of Pharmacy, Muhimbili University of Health and Allied Sciences, P. O. Box 65013 Dar es Salaam, Tanzania; minziobejayesu@gmail.com (O.M.); enali2012@gmail.com (A.K.); 7 College of Medicine and Health Sciences, University of Rwanda, KK 737 Kigali, Rwanda; ebienvenu3@gmail.com (E.B.); mmugisha@nursph.org (M.M.); 8 Department of Pharmacology and Clinical Pharmacy, College of Health Sciences, Addis Ababa University, P.O. Box 9086 Addis Ababa, Ethiopia; eyasumakonnen@yahoo.com; 9 Center for Innovative Drug Development and Therapeutic Trials for Africa (CDT Africa), College of Health Sciences, Addis Ababa University, P.O. Box 9086 Addis Ababa, Ethiopia; 10 Department of Pharmacology and Pharmacognosy, School of Pharmacy, University of Nairobi, P.O. Box 19676-00202 Nairobi, Kenya; olukamarga@yahoo.com (M.O.); anguantai@yahoo.com (A.G.); 11 Pharmacovigilance Centre Lareb, 5237 MH ’s-Hertogenbosch, The Netherlands; e.vanpuijenbroek@lareb.nl; 12 Pharmacovigilance and Clinical Trials, Botswana Medicines Regulatory Authority, P.O. Box 505155 Gaborone, Botswana; partha18@gmail.com

**Keywords:** pharmacovigilance, medicine safety, Neglected Tropical Diseases Program, public health program, East Africa

## Abstract

Monitoring the safety of medicines used in public health programs (PHPs), including the neglected tropical diseases (NTD) program, is a WHO recommendation, and requires a well-established and robust pharmacovigilance system. The objective of this study was to assess the pharmacovigilance systems within the NTD programs in Ethiopia, Kenya, Rwanda, and Tanzania. The East African Community Harmonized Pharmacovigilance Indicators tool for PHPs was used to interview the staff of the national NTD programs. Data on four components, (i) systems, structures, and stakeholder coordination; (ii) data management and signal generation; (iii) risk assessment and evaluation; and (iv) risk management and communication, were collected and analyzed. The NTD programs in the four countries had a strategic master plan, with pharmacovigilance components and mechanisms to disseminate pharmacovigilance information. However, zero individual case safety reports were received in the last 12 months (2017/2018). There was either limited or no collaboration between the NTD programs and their respective national pharmacovigilance centers. None of the NTD programs had a specific budget for pharmacovigilance. The NTD program in all four countries had some safety monitoring elements. However, key elements, such as the reporting of adverse events, collaboration with national pharmacovigilance centers, and budget for pharmacovigilance activity, were limited/missing.

## 1. Introduction

Neglected tropical diseases (NTDs) represent a group of infections caused by a range of viruses, bacteria, protozoa, and parasitic worms that are prevalent in tropical and subtropical regions in 149 countries worldwide. Globally, more than 1.5 billion people suffer from at least one NTD, and over 600 million people live in Africa [1,2,3]. Most of the infected people live in poverty (living on less than US$2 per day), without adequate sanitation and in close contact with infectious vectors, domestic animals, and livestock [2,3,4]. NTDs cause severe disfigurement, disability, or premature death. Annually, more than half a million people die due to NTDs or NTD-related complications [2]. In 2012, the global burden of NTDs was reported to be 56.5 million disability-adjusted life years (DALYs), which is higher than that of malaria (46.5 million) and tuberculosis (34.7 million) [5]. According to the Regional Strategic Plan for NTDs in the African Region, 47 countries are endemic for at least one NTD, and 37 of them are co-endemic for at least five NTDs [6]. The most common NTDs in Sub-Saharan Africa are trachoma, helminth infections, especially soil transmitted helminth infections (STHs), schistosomiasis, and filarial infections, such as lymphatic filariasis (LF) and onchocerciasis [7]. These NTDs can be treated, controlled, and eliminated by preventive chemotherapy (PC) using mass drug administration (MDA) [2,3].

NTDs have been associated with long-term disabilities and adverse consequences, such as hydrocele and/or swelling of the lower limbs or breasts (LF), blindness (onchocerciasis and trachoma), stunting, anemia and malnutrition (STHs), liver and bladder fibrosis, and cancer (schistosomiasis) [8]. Due to the severe suffering and socioeconomic consequences of NTDs, including onchocerciasis, LF, schistosomiasis, and STHs in endemic areas, global programs to eliminate these diseases as public health problems was established by the World Health Organization (WHO). Community- or school-based large-scale distribution of drugs at regular intervals to all at-risk populations without prior diagnosis is WHO’s core intervention strategy to control morbidity and to interrupt transmission, hence stopping the spread of infection and thereby reducing the suffering of the affected poor communities [9,10,11,12]. MDA aims to reduce the burden of selected NTDs, and it has already contributed significantly to improving global health, with the potential for further successes, especially when combined with other interventions, such as sanitation and education programs [8,13,14,15]. Globally, one billion people, including 418 million people in Africa, received treatment for at least one NTD in 2017 [1,16]. The delivery of the PC intervention is usually undertaken by periodic MDA campaigns organized by the NTD public health programs (PHPs) in each endemic country. Given the fact that the mass distribution of drugs to all target populations without prior individual diagnosis is conducted by community drug distributors (CDDs) or schoolteachers with little or no healthcare background, pharmacovigilance to monitor drug safety is vital to boost public confidence in the program.

The drugs used for mass administration deployed under the NTD programs include albendazole or mebendazole for STHs, azithromycin for trachoma, diethylcarbamazine and ivermectin for LF, and praziquantel for schistosomiasis. The medications have a good safety profile when used in a single dose in PC, but mild and transient adverse events (AEs), such as vomiting, drowsiness and dizziness (praziquantel), gastro-intestinal symptoms (ivermectin, albendazole, and mebendazole), fever, skin rashes, muscle ache, orthostatic hypotension, tachycardia, lymphadenopathy, amongst others (ivermectin), following MDA are reported [17]. Rarely, serious adverse events (SAEs), such as hospitalization and death, related to medicines used during MDA campaigns is reported [17]. However, AEs are not always related to the medicinal products, but also other factors, including how the medicinal product is used in the given context. AEs, including SAEs, may occur due to the medications, operational errors, or coincidence [17]. Therefore, without a safety monitoring system in place, it is very challenging to ascertain the cause of an AE/SAE and implement measures to prevent or minimize reoccurrence. Additionally, due to the co-endemicity of NTDs in most geographical regions, concomitant administration of the medications against multiple NTDs is given during MDA to optimize the use of resources, save operational costs, and increase the impact of the interventions [18]. The co-administration of multiple drugs further highlights the need for a rigorous safety monitoring system. Furthermore, in MDA, rare events may occur that will otherwise go undetected without a comprehensive safety monitoring system.

The drugs used for MDA were tested in a population that was different and not comparable to the target MDA population, hence, this is another strong rationale for safety monitoring. It is important for PHPs to identify, assess, quantify, and characterize the risks to individuals and communities. Improvement of practices based on observed problems and learnings should be an integral part of all programs to minimize harm and sustain public confidence in the safety of the medicines used in the NTD programs [19]. A comprehensive pharmacovigilance system within the NTD programs will help detect, evaluate, and prevent AEs.

In Ethiopia, Kenya, Rwanda, and Tanzania, MDA campaigns for the treatment and control of NTDs are organized by the NTD programs that were launched in 2009, 2002, 2008, and 2002, respectively, and since then, the countries have been involved in conducting MDAs for the treatment and prevention of NTDs. However, due to financial and administrative challenges, MDA has not always been implemented regularly per the WHO recommendations. Moreover, safety monitoring has not been given the same prominence as treatment coverage within PHPs, especially in NTD programs, partly because of limited financial resources, among other factors. Therefore, little is known about the safety of the drugs used in PC for NTDs in the community. Even though, globally, approximately one billion people receive MDA for NTDs every year, little attention has been given to research and funding on safety monitoring [1,16,20].

In 2018, 382 million people were exposed to medicines for treatment and control of NTDs in Africa [21]. The safety profile of these drugs may vary depending on the population type, presence of infection, severity of infection, and drug interactions due to co-administration of multiple drugs. The predisposing factors and their influence on AEs can only be established by having a comprehensive pharmacovigilance system in place. MDAs are usually implemented by the NTD programs, so the programs should have pharmacovigilance systems in place to monitor the safety of the medicines. Due to limited scientific research in this area, policy makers, NTD program managers, and healthcare workers are unaware of the safety profile of drugs used in MDAs in Africa. To the best of our knowledge, no studies have evaluated the pharmacovigilance systems within the NTD programs.

The objective of this study was, therefore, to assess and compare the pharmacovigilance systems and practices within the NTD programs in Ethiopia, Kenya, Rwanda, and Tanzania.

## 2. Materials and Methods

### 2.1. Study Design and Assessment Tool

This was a cross-sectional descriptive study assessing and comparing the present pharmacovigilance systems within the national NTD programs in Ethiopia, Kenya, Rwanda, and Tanzania. The East African Community (EAC) Harmonized Pharmacovigilance Indicators tool for PHPs, derived from the WHO pharmacovigilance indicators and the Indicator-Based Pharmacovigilance Assessment Tool (IPAT) [22,23], was used in this study. The indicators are designed to assess the integration of pharmacovigilance related activities in PHPs to identify strengths and limitations. The tool for the assessment of the PHPs contains 20 indicators that address four pharmacovigilance components: (i) systems, structures, and stakeholder coordination; (ii) data management and signal generation; (iii) risk assessment and evaluation; and (iv) risk management and communication. In this study, the assessment was further supplemented with interviews of the NTD personnel and a review of the documentation from the respective NTD programs in order to attain a good understanding of the safety surveillance activities in the program.

### 2.2. Data Collection

The assessment and data collection in the four countries were conducted between July and December 2018. The EAC Harmonized Pharmacovigilance Indicators tool was used as a guide for semi-structured interviews. The assessment team consisted of three individuals, while the respondents were between two to three staff members from the national NTD program, except for Ethiopia, where only one staff member was available for the interview. Apart from the assessment tool, follow-up questions were also asked when deemed necessary until the assessors had gained enough understanding of the availability and functionality of the relevant structure, process, systems, or output/outcome. The responses were recorded on a template developed for data collection for this study, and then the documents were sent back to the respondents for verification. The respondents were pharmacists (in Tanzania and Kenya), a medical doctor, a public health officer (in Rwanda), and a public health officer (in Ethiopia) working for the program.

### 2.3. Comparative Analysis of Results from Individual Country Assessments

The data from the individual country assessments were collated and entered into a template developed for the purpose of this study based on the four pharmacovigilance components of the assessment tool. Tables and a figure were used to present findings of pharmacovigilance performance indicators within the same component.

### 2.4. Ethical Considerations

This study assessed the national pharmacovigilance systems at the national NTD programs in Ethiopia, Kenya, Rwanda, and Tanzania to identify the presence or absence of key pharmacovigilance performance indicators, thereby identifying gaps for future targeted interventions. No personal data were collected, and the informants did not disclose any personal data. Therefore, a waiver of informed consent was requested and obtained from the respective institutional ethics review boards in each participating east African country.

## 3. Results

### 3.1. Systems, Structures, and Stakeholder Coordination

This component of the assessment tool had six indicators, as presented in Table 1. The NTD programs in Ethiopia, Kenya, and Tanzania had a strategic masterplan that included pharmacovigilance-related activities. The NTD programs in Rwanda had a draft strategic plan, which was yet to be approved by the Ministry of Health, but it had components of pharmacovigilance.

As shown in Table 1, none of the countries’ NTD programs had an annual budget specific for pharmacovigilance-related activities. Funding from donors, however, provided financial support for MDA-related materials and activities, including drugs, logistics, and implementation. MDA-related trainings were part of the NTD programs’ tasks, and pharmacovigilance components were included in all four countries. However, pharmacovigilance components were minimally covered.

The NTD programs in all four countries had at least one mechanism in place to disseminate pharmacovigilance information. An information sheet, radio programs, and MDA training were the key mechanisms for the dissemination of pharmacovigilance information. However, none of the NTD programs had a newsletter or information bulletin for dissemination.

- In Rwanda, the NTD program provided healthcare workers with information sheets about the drugs and known AEs associated with the drugs used in MDA. The information sheets were also shared with the community through health centers. Safety information was also included during MDA preparatory meetings and trainings. Additionally, during social mobilization for MDA, the NTD programs addressed issues related to safety on the radio.
- In Ethiopia, the NTD program disseminated pharmacovigilance information during workshops, review meetings, and trainings. The program communicated with the public through the radio services about possible known AEs of the medicines used in MDA and how to report them. However, the program was careful with communicating safety-related issues to minimize misunderstanding and prevent any negative repercussions for the MDA campaign.
- In Kenya, community health extension workers (CHEWs) and CDDs were trained on and briefed about possible adverse drug reactions (ADRs), what to look for, and how to report and address them.
- In Tanzania, like Kenya, the dissemination of pharmacovigilance information was done through MDA training. However, in case of any safety-related issues, the affected person was managed, and information was contained within the area in which the event occurred. Additionally, during community and social mobilization for MDA, the NTD program had a radio program.

The Tanzania NTD program was the only program that had a website with information about the overall program activity, but nothing on pharmacovigilance. Although the Ethiopia NTD program did not have a website, information on the general MDA programs was advertised on the Ministry of Health website, but, again, there was no information on pharmacovigilance.

None of the NTD programs in the four countries had a toll-free number for the public on issues related to the program including pharmacovigilance information. The Ethiopian NTD program shared the national medicine regulatory authority’s (NMRA) toll-free hotline for reporting/seeking medicine safety information. In Kenya, the NTD program shared the NMRA hotline number with the MDA trainees. However, this was initiated for the first time in 2018. In Rwanda, the NTD program did not have a publicly advertised toll-free number or phone line. People were advised to contact the local health centers if they suspected that they may be suffering from AEs. In Tanzania, the NTD program did not have a toll-free number, however, the program distributed leaflets to the community with the contact information of the program for those who sought more information about the program, but this is not specifically for pharmacovigilance. The Tanzania NTD program depended on the NMRA to provide medicine safety and pharmacovigilance information.

In Rwanda, if there was a critical concern, then the national team would be notified through the campaign supervisors. The national program worked in close collaboration with district hospital supervisors in charge of the MDA campaign (a WhatsApp group has been created to facilitate communication). Like in Tanzania, the NTD program may not know about all SAE- or AE-related hospitalization if the cases were handled at the health center and hospital level when they were not reported. The Kenyan NTD program did not receive individual case safety reports (ICSRs), but AEs were reported, handled, and dealt with at the level at which they occurred.

**Figure 1:**
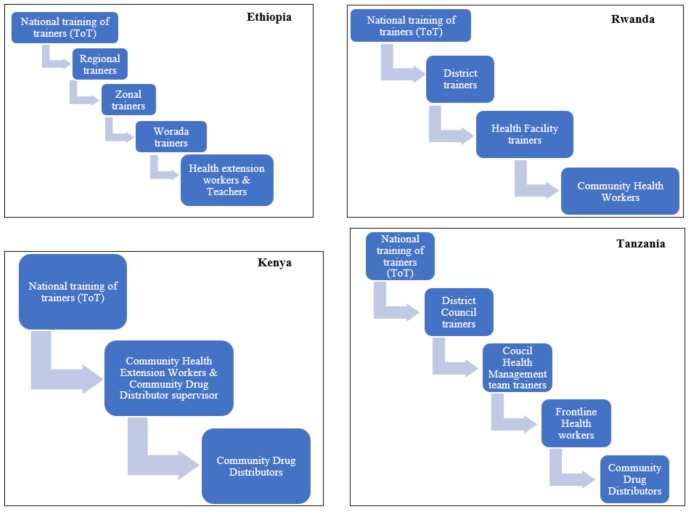
Pre-MDA cascade training model by country NTD program. ToT, training of trainers.

All of the NTD programs in the four countries used the training of the trainers’ cascade model, as shown in Figure 1. The trainings were mainly focused on the implementation and coverage of MDA, but a small component of medicine safety as part of the pre-MDA trainings was given. In all four countries, the number of personnel trained during the last 12 months is presented in Table 2.

Regarding NTD treatment guidelines, in Ethiopia and Tanzania, the NTD program treatment guidelines provided information and guidance on pharmacovigilance activities. The Ethiopian NTD program treatment guideline was developed in collaboration with the NMRA, Ethiopian Food and Drug Authority (EFDA), and other partners. The guideline is updated yearly, and the most recent one was the 2018 version. Rwanda had a draft NTD program treatment guideline that also included instruction for pharmacovigilance activities. The NTD program in Kenya used the WHO NTD treatment guidelines.

The NTD programs in Ethiopia, Kenya, Rwanda, and Tanzania reported that they would consider evidence on safety data when developing/updating standard treatment guidelines. However, local safety data were limited.

### 3.2. Data Management and Signal Generation

There was only one indicator of this component of the assessment tool, which was the AE reporting tool used by the NTD program. In Ethiopia and Tanzania, the NTD programs used the national ADR reporting forms of their respective NMRA. In Kenya, the NTD program gave healthcare workers alternatives: to either report using the NMRA reporting form or the WHO generic form. The Rwandan NTD program used the national AE reporting form adapted from the WHO.

### 3.3. Risk Assessment and Evaluation

Eight indicators were assessed in this component of the assessment tool, i.e., AE information (six indicators), active surveillance (one indicator), and collaboration on risk management (one indicator). Zero ICSRs were received by the NTD programs in Ethiopia, Kenya, and Tanzania in the last 12 months (2017/2018), despite the large number of individuals that received MDA, as presented in Table 3. According to the Ethiopian NTD program, there were three deaths related to praziquantel and azithromycin in 2015/2016. The Kenyan NTD program did not collect ICSRs, and AEs were reported, handled, and dealt with at the level at which they occurred if they were observed. In Rwanda, the NTD program received 42 reports from health facilities during the campaign from all 30 districts in 2017/2018. This was a summary (aggregate) of the number and type of AEs per hospital catchment area, not ICSRs, and such summary reports were not reported to the national pharmacovigilance center, partly because the national pharmacovigilance system in Rwanda was not fully functional at the time. In Tanzania, all AE and SAE reports were meant to be sent directly to the NMRA and not to the NTD program, but no reports were received in 2017/2018.

The number of suspected product quality issues detected through the NTD programs in the last 12 months was zero in all four countries. In Kenya, the NTD programs assumed that the products were of good quality, as they were from the WHO pre-qualified manufacturing companies. The Rwandan NTD program did not have access to a quality control laboratory to check the quality of drugs. In the last 12 months, the number of medicine-related hospital admissions of individuals exposed to medicines in the NTD programs in all four countries were unknown. The Rwandan NTD program mentioned that some hospital admissions related to praziquantel administration, reported through the campaign supervisory channel, were investigated by the district and hospital team under the coordination of the national NTD team. However, no ICSRs on the abovementioned cases were recorded and submitted to the program or national pharmacovigilance center. Additionally, cases handled and managed at the health center and hospital level were not always communicated/reported to the national NTD programs.

None of the four NTD programs had conducted an active surveillance safety/efficacy study in the last three years. Only a storage condition study was conducted by the Kenyan NTD program.

In Ethiopia, the NTD program and national pharmacovigilance center collaborated for the planning and development of national guidelines, as well as pre-MDA trainings. There were no regular meetings between the two institutions, but they met when there was a need. In Kenya, Rwanda, and Tanzania, the NTD programs and the respective national pharmacovigilance centers hosted at the respective NMRAs did not communicate on risk management plans.

### 3.4. Risk Management and Communication

There were five indicators in this component of the assessment tool, including: (i) the average time between the identification of safety signal to communication to stakeholders, (ii) the presence of a program-related newsletter, (iii) the number of requests on the safety of medicine received in the previous calendar year (2017/2018), (iv) the number of medicine safety issues identified from outside sources acted on locally in the previous calendar year (2017/2018), and (v) the number of public or community education on medicine safety in the previous calendar year (2017/2018).

In Ethiopia and Kenya, the NTD programs did not have a specified time lag between the identification of safety signal/SAE/significant medicine safety issues generated nationally and communication to health care workers and the public. The NTD programs did not have a standard procedure in place for this. In Rwanda and Tanzania, the NTD programs reported an average lag time between 24 h and one week, depending on the severity/seriousness of the AE, but this was not documented, hence, it could not be verified.

None of the four countries’ NTD programs had a program-related newsletter that featured ADRs observed or medicine safety information in general. Neither had they received/recorded requests for information about medicine safety. In Ethiopia and Tanzania, information about medicine safety was said to be discussed during trainings, supervisory visits, and/or review meetings. In Rwanda, it was reported that the national NTD program staff received calls about medicine safety from the public, but this was not recorded. Initially, the program used to receive many calls when the MDA interventions were newly introduced. That phase has died out, and currently, they receive approximately 10 calls/year on medicine safety. The staff addressed all of the calls they received in the last 12 months (2017/2018). In all four countries, the NTD programs did not act on any medicine safety issues identified from outside sources in the previous 12 months (2017/2018), and, according to their knowledge, no safety issues were identified.

Regarding public or community education activities relating to medicine safety, although the NTD programs in all four countries carried out a pre-MDA campaign, community mobilization and sensitization activities were focused on program implementation. In Ethiopia, local media, village criers, community leaders, and a health development army are used for these activities. Medicine safety is covered during sensitization campaigns in Ethiopia and Tanzania. In Kenya, safety monitoring is not given prominence during these sensitization campaigns. In Rwanda, prior to drug administration, an education session is provided to the community (mainly for praziquantel) through radio, village criers, schools, churches, and cars mounted with speakers. The radio sessions included a question and answer session, where questions on medicine safety from the public were addressed. The NTD program also had adverts that included medicine safety information. These public/community activities on medicine safety were irregular, partly due to the lack of enforcement of MDA guidelines.

## 4. Discussion

This study assessed and compared the pharmacovigilance systems within the respective NTD programs in Ethiopia, Kenya, Rwanda, and Tanzania to provide an overview of the current status, identify gaps, and provide recommendations for targeted interventions. The EAC Harmonized Pharmacovigilance Indicators tool derived from the WHO pharmacovigilance indicators and the IPAT [22,23] was used in this assessment.

Our findings indicate that all of the NTD programs in the four countries had an operational document, such as a strategic master plan and treatment guidelines, that included pharmacovigilance, however, the Rwanda NTD strategic plan was in the form of a draft pending approval from the Ministry of Health at the time of data collection. The Rwanda NTD strategic plan has now been approved. Additionally, all of the NTD programs in the four countries have AE reporting forms, either the national reporting form or the WHO generic form. The availability of such documents highlights the NTD programs’ commitment to medicine safety monitoring [25].

The main strength of the NTD programs in all four countries was the inclusion of a medicine safety component during pre-MDA campaign trainings for healthcare workers and drug distributers. This information indicates that pre-MDA preparatory activities include the planning and management of safety during MDA. However, this is not reflected in the number of ICSRs and AEs that the NTD programs received or reported to the respective NMRAs. For example, in the last calendar year (2017/2018), both NTD programs and the respective NMRAs in all four countries received zero ICSRs following MDA. This may indicate that information on the medicine safety component was either limited or not sufficiently addressed during the pre-MDA trainings, or there was a lack of follow-up after completion of the MDA implementation process. Therefore, there is a need for more emphasis on medicine safety monitoring and reporting of AEs as recommended by the WHO [17].

Another strength noted was that the NTD programs in all four countries had mechanisms to disseminate pharmacovigilance information. The main mechanisms were pre-MDA trainings and radio programs. Although the cascade model for training used by all four countries is cost-effective, it is well-known that such a long chain (3–4 steps) of information sharing also carries a risk of distortion of the message [26]. No effort of ensuring the accuracy of the information reaching the community was presented. Additionally, mechanisms such as a newsletter, bulletin, or information sheets were not used as a means of communication, and only the Rwanda NTD program had an information sheet including medicine safety for healthcare workers. These mechanisms are cost-effective, and can be useful especially in countries with limited resources. Furthermore, websites can also be used to disseminate medicine safety information. Only the Tanzania NTD program had a website, though it had no pharmacovigilance information. Incorporating safety information and guidance on how to report AEs on the website would be valuable for all stakeholders, including the public. During the dissemination activities, the communities should be educated about the expected mild reactions and, for any unusual severe or serious symptoms, they should seek medical attention at a health facility [27], although many PHPs, including the NTD programs, are concerned that discussing potential AEs may have a negative impact on the treatment coverage. However, a systematic review investigating the facilitators and barriers of MDA for LF in sub-Saharan Africa reported “awareness creation through innovative community health education programmes” as a facilitator [28].

At present, the identification and reporting of MDA-related harms are not being captured within the respective NTD programs in all four countries. When many millions of people are exposed to drugs annually, both mild and serious AEs temporally related to treatment are likely to occur. However, in all four countries, there was no established system to identify and record such events or to establish whether they might be treatment-related or coincidental. Furthermore, there is no available data to determine whether mass treatment is safe under field conditions or not. Pharmacovigilance following MDA in NTD programs in all of the four countries is as neglected as the diseases. No proper follow-up assessment of individual benefit/harm balance or active surveillance study to determine the safety and effectiveness of the medicines given in MDA are conducted. Moreover, in all four countries, there was no standard procedure for identification of the safety signal/serious AE/safety issue/to communication with the healthcare workers and the public (if necessary). Although Tanzania and Rwanda reported that the lag time was between 24 h and one week depending on severity of AEs, no documentation or standard operating procedure was available for verification.

Resources for the ensuring of medicine safety during community MDA is limited. There was no specific budget allocated for pharmacovigilance activities, and this may have contributed to the underreporting of AEs. To implement the pharmacovigilance components in the masterplan and treatment guidelines, thereby monitoring medicine safety, the NTD programs need to have a specific budget for pharmacovigilance, as reported by other PHPs, such as malaria, HIV/AIDS, TB, and immunization programs [25]. None of the countries’ NTD programs had a toll-free number available to the public for the reporting of AEs and inquiries related to other medicine safety issues. However, in Ethiopia and Kenya, the NTD programs shared the hotline number of their respective NMRAs, and this can be adapted in Tanzania and Rwanda.

A recent study reported that the national pharmacovigilance centers in Africa found it challenging to engage PHPs in a sustainable way due to several factors, including limited collaborations [29]. Our findings also show that there is limited collaboration between NTD programs and their national pharmacovigilance centers hosted at the respective NMRAs. Only the Ethiopia NTD program had collaborated with the NMRA on the development and review of the treatment guidelines and pre-MDA training. The national pharmacovigilance centers hosted at the NMRAs and NTD programs should collaborate to develop guidelines for safety surveillance of drugs used in MDA under the programs, jointly design and train healthcare workers during MDA preparations, review treatment guidelines, and carry out active safety surveillance when new therapeutic regimens are introduced. The NTD programs and national pharmacovigilance centers should plan and conduct the abovementioned pharmacovigilance activities to avoid duplication of efforts and optimize the use of resources [23]. Collaboration with other partners, such as academic institutions and researchers, can further strengthen safety monitoring by conducting pharmacovigilance studies of public health importance [25,30]. Such collaborative safety surveillance would provide information on the safety of the treatment regimens, the true incidence of ADRs, the risk factors, and the tolerance in patients at risk for ADRs. It would demonstrate that the well-being of the target population receiving treatment in the communities is important to the NTD programs. So far, no observational active surveillance studies on AEs following MDA have been carried out by any of the NTD programs in the four countries.

In all four countries, the NTD programs inform healthcare workers to report suspected AEs directly to the health facility/NMRA. This further emphasizes the need for a collaboration between NTD programs and their respective NMRAs to keep the NTD programs informed on the number of AEs associated with the medicines used in MDA.

On the other hand, the respective countries’ NMRAs should give feedback to the NTD programs on the number of ICSRs received by the national pharmacovigilance programs and safety profiles of the medicinal products used in MDA. Zero ICSRs associated with medicines used in MDA for targeted NTDs were submitted in the last calendar year (2017/2018). In 2017/2018, the majority of ICSRs that were submitted to the national pharmacovigilance center in Ethiopia and Kenya were from the tuberculosis and HIV/AIDS programs [30]. Unlike the malaria, tuberculosis, and HIV programs, drugs in NTD programs are given through MDA to all at-risk populations without prior diagnosis or screening, with no possibility of post-MDA follow-up for timely identification and management of AEs. Therefore, proper integration of pharmacovigilance activity in MDA planning and implementation activity is vital to boost public confidence in the program to increase MDA adherence.

In 2006, WHO reported that, in most developing countries, there were insufficient resources within the public health system, including PHPs, for competence development, capacity building, and monitoring the efficacy and safety of medicines [31]. The major parts of available resources are often concentrated on developing PHP capacity to reduce disease morbidity and mortality [31]. In this study, there were limited or no resources for capacity building for monitoring drug safety. Therefore, competence development and capacity building in pharmacovigilance is urgently needed within the NTD programs in all four countries.

To improve the current status of the pharmacovigilance systems within the NTD programs, strong collaboration and coordination with all stakeholders, including the national pharmacovigilance center of the respective NMRA, is crucial. Engagement of pharmacovigilance experts in providing training of CHEWs, CDDs, and schoolteachers on how to prevent, monitor, manage, and report suspected AEs following MDA during the MDA campaign is important. Providing relevant information for the community on the PHP’s website about known AEs and their associated risk factors, as well as how to manage and report MDA-associated AEs, would boost public confidence in the program. Budget allocation for pharmacovigilance activities in the PHP program is key to improve the identified gaps. As the number of new drugs, vaccines, and interventions being introduced into low- and middle-income countries is on the rise, safety and efficacy monitoring in the local indigenous target population is critical to identify and prevent rare AEs.

Overall, most of the information captured in this study was verified using operational documents, including strategic masterplans and guidelines. However, it was not possible to verify all of the information gathered through structured interviews and discussions with representatives (interviewees) of the national NTD program, and there we relied on the respondents’ accuracy of gathered information.

## 5. Conclusions

All four countries had started to integrate pharmacovigilance within their NTD programs, and a strategic master plan and treatment guidelines, including pharmacovigilance components, an AE reporting form, and mechanisms to disseminate medicine safety information, are in place. However, key elements to monitor safety, such as the reporting of AEs, specific budget for pharmacovigilance, sustainable collaboration between the NTD programs and the national pharmacovigilance centers (NMRAs), communication with other stakeholders, and pharmacovigilance capacity building are limited/missing. Our finding highlights key gaps for targeted intervention to promote public medicine safety and increase the success of the NTD programs in Ethiopia, Kenya, Rwanda, and Tanzania.

## Figures and Tables

**Table 1:** National systems, structures, and stakeholder coordination.

	Indicators					
Country	Pharmacovigilance Activities Included within the Strategic/Operational Plans	Mechanism to Disseminate Pharmacovigilance Information	Toll Free Number	Treatment Guidelines/Protocol Considering Pharmacovigilance	Budget for Pharmacovigilance	Website
Ethiopia	✔	✔	✔ *	✔	X	X
Kenya	✔	✔	✔ *	✔	X	X
Rwanda	✔ ^#^	✔	X	✔	X	X
Tanzania	✔	✔	X	✔	X	✔

✔ = Present; X = missing/not available; ^#^ draft strategic master plan (not approved); * refer to the national pharmacovigilance center number.

**Table 2:** The number of healthcare workers trained in pre-MDA training in the last 12 months (2017/2018) per country NTD program.

Country (NTD Program)	Number of Healthcare Workers That Received MDA Training That Included Pharmacovigilance Sensitization in the Last 12 months (2017/2018)	Country’s Population Size (2018) ^
Ethiopia	42 Regional trainer of trainers 272 Zonal trainers 3200 Woreda trainers 36,000 Health extension workers 50,000 Teachers	109,224,414
Kenya	79 Trainer of trainers 393 Community health extension workers 7850 Community drug distributors	51,392,565
Rwanda	248 District and hospital NTD team members 1477 Health center NTD team members 42,000 Community health workers	12,301,970
Tanzania	1080 Regional and district NTD team members 6195 Frontline health workers 46,070 Community drug distributors 21,807 Teachers	56,313,438

NTD neglected tropical disease. ^ The population size for each country was taken from Worldometers [24].

**Table 3:** Number of individuals that received MDA under the NTD program during the last 12 months (2017/2018).

Country (NTD Program)	Number of Individuals That Received MDA under the NTD Programs during the Last 12 Months (2017/2018)	Country’s Population Size (2018) ^^^
Ethiopia	7 million (Schistosomiasis) 5 million (Lymphatic Filariasis) 14 million (Onchoceriasis) 20 million (Soil Transmitted Helminths) > 15 million (Trachoma)	109,224,414
Kenya	3,017,897 (Lymphatic Filariasis) 395,962 (Trachoma) 521,643 (Schistosomiasis) 6,360,900 (Soil Transmitted Helminths)	51,392,565
Rwanda ^§^	1,154,486 (Schistosomiasis) 4,851,720 (Soil transmitted Helminths, First Round) 5,060,034 (Soil transmitted Helminths, Second Round)	12,301,970
Tanzania	6,813,959 (Schistosomiasis) 8,289,807 (Lymphatic Filariasis) 5,150,732 (Onchocerciasis) 9,669,728 (Soil transmitted Helminths) 1,724,950 (Trachoma)	56,313,438

MDA, mass drug administration. ^^^ The population size for each country was taken from Worldometers [24]. ^§^ In Rwanda, the NTD program carried out MDA only for schistosomiasis and soil transmitted helminths, and MDA for soil transmitted helminths was carried out biannually.
